# An Automated, Contamination-Controlled VQA Benchmark for Evaluating Vision-Language Models on 3D Oncology Imaging

**DOI:** 10.21203/rs.3.rs-10747614/v1

**Published:** 2026-09-11

**Authors:** Bo Liu, Hanxue Gu, Xiangru Li, Zheren Zhu, Jacob Ellison, Kang Wang, Ke Sheng, Steve Braunstein, Janine Lupo, Yang Yang, Hui Lin

**Affiliations:** University of California, San Francisco; University of California, San Francisco; University of California, San Francisco; University of California, San Francisco; University of California, San Francisco; University of California, San Francisco; University of California, San Francisco; University of California, San Francisco; University of California, San Francisco; University of California, San Francisco; University of California, San Francisco

## Abstract

Vision-language models (VLMs) are increasingly applied to medical imaging, yet public benchmarks may reward memorization over perception: their images and questions can enter pretraining corpora, and many items remain answerable from question text alone. We present an automated, agent-driven pipeline that builds multiple-choice benchmarks directly from paired private radiology reports and three-dimensional oncology imaging. It generates two complementary question types: schema-driven items populated deterministically from established reporting frameworks, and report-derived items verified against the source text. Because the source reports are private and single-institution, the resulting questions cannot have entered any model’s pretraining data, controlling instance-level contamination by construction. We applied the pipeline to four oncology cohorts (liver CT, liver MRI, lung CT and brain MRI; 2,509 cases and 33,870 questions) and evaluated five contemporary VLMs zero-shot. Accuracies ranged from 0.28 to 0.81 and no model was reliable across cohorts. Replacing the image with a blank input changed accuracy little for the highest-scoring models, and on schema-driven brain questions frontier models scored 0.17–0.19 higher on public than on matched private imaging. The benchmark thus separates image-dependent performance from question-text and dataset-familiarity effects, and the released pipeline lets institutions regenerate it on their own reports and images.

## Introduction

Vision-language models (VLMs) are increasingly proposed for clinical imaging, yet whether they can be trusted to read oncology studies remains poorly characterized. To be considered trustworthy, an evaluation must be clinically relevant, statistically robust, and free from confounding variables. Current medical visual question-answering (VQA) benchmarks rarely combine all three of these essential qualities. Manually annotated datasets (VQA-RAD^1^, PathVQA^2^, SLAKE^3^) are clinically curated but small, whereas large web- or literature-sourced sets (OmniMedVQA^4^, PMC-VQA^5^) scale up but enter VLM pretraining corpora, so high scores may reflect memorization rather than perception. Domain-adapted biomedical VLMs, including Med-PaLM^6^ and LLaVA-Med^7^, established the potential of domain-specific multimodal adaptation. MIRAGE^8^ recently showed that VLMs answer public medical VQA with little accuracy loss when the image is removed, implicating language priors and data leakage. Oncology imaging compounds the difficulty: studies are volumetric, so a 2D-input VLM must answer questions using information distributed across multiple slices of an imaging acquisition, which radiologists interpret by reviewing slices and sequences collectively, and every case is a confirmed cancer patient, shifting questions from “is there an abnormality” to lesion characterization, disease extent, and treatment-relevant features. Our objective in this work was therefore not to construct an exhaustive model leaderboard, but to determine whether a renewable benchmark generated from private reports can distinguish image-dependent performance from question-text and dataset-familiarity effects.

## Results

### An automated, contamination-controlled benchmark

To evaluate VLMs under these constraints, we built an automated, agent-driven pipeline that generates multiple-choice VQA directly from paired private radiology reports and 3D images (Fig. 1). It produces two complementary question types. RADS-style questions are populated deterministically from a clinician-reviewed schema of visual findings grounded in established reporting frameworks (LI-RADS^9^, Lung-RADS^10^, and a RANO-aligned brain schema^11^), with limited language-model calls at generation time and therefore standardized. Report-derived questions are generated by a language model from the radiologist’s findings and impression, then filtered by a report-grounded check that retains only questions whose answer is explicitly supported by the source text. By utilizing private, single-institution source reports, we guarantee that the generated questions remain isolated from any model's pretraining data, inherently preventing instance-level contamination. Furthermore, the pipeline is highly automated, requiring human intervention only for schema and template approval. Full details are in Methods.

### Zero-shot accuracy across four oncology cohorts

We applied the pipeline to four oncology cohorts: liver CT, liver MRI, lung CT, and brain MRI, and evaluated five contemporary VLMs zero-shot. Selected from systems available through commercial APIs or open-weight releases, the panel spans both general-purpose and medically adapted model classes: two frontier closed models (Claude Opus 4.6 and GPT-5.2), one open-weights frontier model (Qwen3-VL-30B), and two purpose-built medical specialists at different scales (MedGemma1.0-27B, MedGemma1.5-4B)^12^. Models received only the image, question, and options, never the report. No model is reliable across cohorts, and none dominates (Table 1): accuracies range from near the random baseline to 0.81, and the ordering of models differs from cohort to cohort. The medical specialist MedGemma1.0-27B has the highest point accuracy in both liver CT cells, Qwen3-VL-30B the highest on lung RADS-style, and Claude Opus 4.6 the highest on liver MRI, lung report-derived, and both brain cells, reaching 0.81 on brain report-derived. Yet that same specialist reaches only 0.47 on brain, a lower accuracy than every general-purpose model, and on liver MRI Qwen3-VL-30B does not exceed its random baseline (0.295 ± 0.016 vs baseline 0.291), consistent with limited transfer of current 2D-VLM pretraining to contrast-rich, multi-sequence MRI.

### Cohort difficulty varies by model class

Cohort difficulty splits by model class. Brain MRI yields the highest accuracies for the frontier models: Claude Opus 4.6 and GPT-5.2 exceed 0.75 on report-derived questions, but the medical specialist MedGemma1.0-27B scores below 0.47 there, lower than its liver CT accuracy, showing that performance varies substantially across imaging cohorts even within a medically adapted model. Liver MRI has the lowest mean accuracy of any cohort: clinical reading integrates information across multiple sequences, which the model must reconcile from separate composite images rather than from a co-registered volume. Liver CT is hard for a different reason, intrinsically low lesion-to-parenchyma contrast that demands fine-grained discrimination even within one slice. The effect of question type varies with model class rather than being uniform: the frontier models score higher on report-derived items, the MedGemma specialists show no consistent preference, and Qwen3-VL-30B is the only model whose accuracy is higher on RADS-style than on report-derived, doing so on the liver and lung cohorts but not on brain MRI.

### Accuracy when the image is removed

A benchmark score is meaningful only if it reflects image reading. To separate visual evidence from language-prior shortcuts, we re-ran one model from each class, the two closed frontier models (Claude Opus 4.6, GPT-5.2) and the larger medical specialist (MedGemma1.0-27B), with the image replaced by a blank input, scoring each model on the question set shared by its sighted and blind runs (Table 2). The sighted-minus-blind drop Δ depends on both model and cohort. The largest sighted-minus-blind differences occur in only a few cells: MedGemma1.0-27B loses 0.29 on liver CT report-derived and GPT-5.2 loses 0.12–0.16 on liver MRI. The highest-scoring model also changes little after image removal: Claude Opus 4.6’s Δ falls entirely within the pre-specified ± 0.05 equivalence margin on every cohort, including liver (liver CT Δ = −0.07 and + 0.01; liver MRI Δ ≈ 0), showing that its performance changes little when visual input is removed. Lung CT accuracy is essentially unchanged when the image is removed for both frontier models (GPT-5.2 Δ = −0.016 and − 0.031; Claude even − 0.16 on RADS-style, blind 0.73 versus sighted 0.56), a pattern compatible with substantial question-text signal; this analysis cannot determine whether that signal arises from pretraining exposure or other text-side cues. Thus, a high benchmark score can coexist with little change after image removal.

### Public versus private brain imaging under matched generation

Brain MRI raises a sharper question. Because its public-cohort imaging (PDGM^13^) could be familiar from pretraining, we asked whether brain accuracy reflects tumor reading or image-distribution memorization by repeating the protocol on a second, fully private in-house brain cohort generated with the identical pipeline, and compared the two with matched question generation (Table 3). On report-derived questions, the two cohorts are indistinguishable within the reported intervals, and every model’s blind accuracy falls within the ± 0.05 margin of its sighted accuracy, and the frontier models stay high even when blind (Claude near 0.81, GPT-5.2 near 0.74), showing that report-derived brain accuracy changes little when visual input is removed. The large public-only sighted-minus-blind difference suggested by an earlier, generation-mismatched comparison disappears under matched generation. A difference remains on RADS-style; however, the frontier models score 0.17 to 0.19 higher on public than private imaging (sighted: Claude 0.67 versus 0.50, GPT-5.2 0.65 versus 0.46), whereas MedGemma1.0-27B differs by < 0.01. The sighted-minus-blind patterns differ by imaging source: blinding raises Claude’s private RADS accuracy by 0.133, whereas on public imaging sighted accuracy is similar to or higher than blind accuracy (GPT-5.2 Δ = 0.09). This pattern is compatible with an influence of image-source familiarity, but the present analysis does not establish its cause or distinguish familiarity from other cohort differences. The blind condition also indicates available nonvisual signal: Brain RADS-style questions are predominantly binary (≈ 92% yes/no), and the blinded frontier models exceed the random baseline by up to 0.17, which may reflect skewed clinical base rates or other question-text cues; MedGemma1.0-27B does not exceed its baseline and yields a near-baseline estimate of brain-RADS performance. Contamination conclusions are thus only as trustworthy as the consistency of the benchmark that produces them.

### Per-subcategory failure patterns

A per-subcategory breakdown of report-derived questions (Fig. 2) resolves where the aggregate accuracies come from. In every cohort the lowest-accuracy subcategory, pooled across the five models, is a quantitative or graded judgment: lesion size on liver CT (0.295) and on liver MRI (0.276), Lung-RADS category on lung CT (0.317), and mass-effect grading on brain MRI (0.481). Because subcategories differ in their option-count mix, raw accuracies are not directly comparable across them, so each is also referenced to its own chance level (Supplementary Table 2). Those four subcategories clear chance by only + 0.093, + 0.071, + 0.067 and + 0.190 respectively, whereas qualitative recognition subcategories clear it by far wider margins: anatomic distribution on liver CT (+ 0.467), pleural findings (+ 0.493) and clinical interpretation (+ 0.522) on lung CT, and oedema on brain MRI (+ 0.515). The pattern is a property of the task rather than of any one system: across the five models, accuracy on liver CT lesion size spans only 0.25 to 0.35 and on Lung-RADS category 0.28 to 0.41, so no model escapes it. Recognising that a finding is present is therefore largely within reach, whereas assigning it a size, an ordinal category or a severity grade is not, even when the relevant region is visible and the same model answers recognition questions on the same images well.

### Multiplicity-controlled confirmation

The intervals in Tables 1 to 3 are pointwise and 76 of them are reported, so before any individual contrast is relied upon we repeated the same case-clustered resampling at higher resolution and controlled the false discovery rate within two families (Methods). Of the 40 accuracy-versus-chance tests, 37 remain above chance after Benjamini-Hochberg correction. The three exceptions are the near-baseline cells: Qwen3-VL-30B on liver MRI for both question types, and MedGemma1.0-27B on brain RADS-style, so those observations are not artefacts of a single unadjusted interval (Supplementary Table 1).

The 30 unique image-effect cells resolve completely (Table 4): 17 show a Δ that differs from zero after FDR control, 16 are equivalent to zero within the prespecified ± 0.05 margin by two one-sided tests, and 3 satisfy both, being reliably nonzero yet smaller than the margin. No cell is left undetermined, so at this margin the design distinguishes a genuine image effect from its absence in every condition tested.

The resolved pattern sharpens the main finding rather than softening it. For Claude Opus 4.6, the highest-scoring model, all five report-derived cells are equivalent to zero, and its only three FDR-significant effects are negative and all RADS-style: on liver CT (Δ = −0.065), lung CT (−0.163) and private brain (−0.133), removing the image improved accuracy. MedGemma1.0-27B shows the opposite profile, large positive effects on liver CT (+ 0.102 RADS-style, + 0.291 report-derived) but all four brain cells equivalent to zero, two of them reliably nonzero yet still inside the margin. GPT-5.2 lies between the two, with positive effects on both liver cohorts and on public-brain RADS-style (+ 0.092) and equivalence on lung RADS-style and on brain report-derived questions in both cohorts.

## Discussion

Our objective was not to construct an exhaustive model leaderboard, but to determine whether a renewable, privately generated benchmark can distinguish image-dependent performance from question-text and dataset-familiarity effects; the five-model panel is a snapshot of contemporary systems rather than a comprehensive survey, and the per-cohort orderings reported here should not be read as definitive rankings. Read with that scope in mind, these results carry a direct message for clinical adoption: current VLMs, including the medical specialists tested, are not yet reliable readers of volumetric oncology studies presented in the reduced 2D formats such systems accept, and headline accuracies may overstate visual competence wherever the question text is solvable from priors: lung CT, and brain MRI for both public and private imaging, while comparatively large sighted-minus-blind differences are observed on liver for MedGemma1.0-27B and GPT-5.2 (all such differences remain significant under Benjamini-Hochberg FDR control, and the small brain and lung report-derived differences are confirmed equivalent to zero within ± 0.05 by two one-sided tests; Table 4). The subcategory pattern is consistent with this input constraint: size, ordinal category and severity grading all rest on spatial measurement, which survives the montage reduction poorly, whereas presence-or-absence recognition survives it well. The reporting tasks that turn on measurement, size tracking for treatment response, Lung-RADS assignment, mass-effect grading, are thus the least likely to transfer to current 2D-input systems, and they are also the ones that most directly drive management decisions. An alternative reading of the blind-ablation result must be addressed: at roughly 128 × 128 pixels per tiled slice, the montage itself may erase the visual evidence, so that blind-equivalent accuracy reflects an impoverished input rather than a model property. Three observations argue against rendering as the sole explanation. First, the montage demonstrably carries exploitable signal: MedGemma1.0-27B gains 0.291 on liver CT report-derived questions from the same composites. Second, the failure profile is not uniform degradation but a systematic dissociation, presence-versus-absence recognition clears chance by wide margins on the same images on which size, ordinal category and severity grading barely clear it, which is the signature of resolution-limited measurement, not of an uninformative image. Third, where the image does change behavior it can do so in either direction, including the three conditions in which removing the image significantly improved Claude Opus 4.6's accuracy. The montage is therefore best understood as part of what is being evaluated: it is the input format that 2D-interface systems impose on volumetric studies today, and the benchmark measures what survives it.

Two properties of that confirmatory analysis matter for how benchmark results should be read. An FDR-controlled test can establish that an effect exists but never that one is absent, and the claims that carry this paper are null-direction claims: that accuracy changes little when the image is removed. They are supported here by equivalence testing against a margin fixed in advance, not by a failure to reach significance, an inference that is not available from an unadjusted interval that merely overlaps zero, though benchmark reports commonly draw it. The three cells that are simultaneously significant and equivalent show why both procedures are needed, since a difference can be statistically reliable and still too small to matter. The equivalence counts are conditional on the ± 0.05 margin we fixed before the analysis; a stricter margin would move cells from equivalent to undetermined without changing which effects are real. Several limitations are inherent. 2D-input models receive each sequence as one 12 × 12 composite image of 144 axial slices rather than as a navigable volume, so an individual slice is rendered at no more than roughly 128 × 128 pixels, understating what slice-aware or 3D-native systems (M3D^14^, RadFM^15^, Merlin^16^) might achieve. Report-derived questions, though grounded, can carry residual language-model authoring bias, and their answer keys were validated by an automated report-grounded check with human review at the schema and template level rather than by per-item radiologist adjudication. All cohorts originate from a single institution, so reporting conventions and case mix may not generalize. The blind ablation covers three of the five models, so visual-reliance conclusions are restricted to those systems. Finally, LI-RADS and Lung-RADS were developed for at-risk screening and surveillance populations rather than cohorts of confirmed cancer patients; we use them here as schemas of visually assessable findings, not as clinical category assignments. Contamination-controlled evaluation thus needs private reports and a generation process held fixed across the cohorts being compared, so that apparent visual signals are not generation artifacts. We release the pipeline as an open agent skill so any institution can rebuild a modality-specific, contamination-controlled benchmark on its own paired reports and images, optionally with a locally hosted language model, turning evaluation from a static public artifact into a private, renewable in-house test.

## Methods

### Study design and cohorts

We applied the pipeline to four oncology cohorts: brain MRI, liver MRI, liver CT, and lung CT, in which every case is a confirmed cancer patient. The brain MRI cohort is a hybrid configuration: imaging derives from the publicly available UCSF Preoperative Diffuse Glioma MRI (PDGM) cohort^13^ paired with private institutional reports; the other three cohorts are fully private from the same institution. Case and question counts are given in Table 1. A fifth, fully private in-house brain MRI cohort is generated identically but reserved for the public/private contamination ablation (Table 3), not the main benchmark. For 2D-input models, each imaging sequence is rendered as a single composite image: 144 axial slices are sampled uniformly across the volume and tiled into a 12 × 12 montage, so that a sequence is presented to the model as one composite image rather than as a navigable volume. Questions that require more than one sequence receive one composite image per required sequence. CT series use organ-appropriate intensity windowing, and each composite is resized so that its longest dimension does not exceed 1,536 pixels, leaving at most roughly 128 × 128 pixels per tiled slice.

### Benchmark construction: RADS-style questions

For each dataset, a schema of clinically relevant visual features is induced from a stratified sample of reports and grounded in established reporting frameworks (LI-RADS^7^ for liver, Lung-RADS^8^ for lung, and an institutional RANO-aligned schema^9^ for brain). Each field corresponds to a finding directly assessable from the image, and clinicians review the schema for visual answerability before generation. Fields are populated deterministically from report text (binary fields map to yes/no; multi-value fields to 3–5 options with dataset-wide distractors).

### Benchmark construction: report-derived questions

Report-derived questions are generated by prompting a language model with the parsed findings and impression sections to produce multiple-choice questions spanning the radiologist’s clinical content. Report-derived items contain two to five answer options, with four-option items predominating; the option range and predominant four-option configuration are shown in Fig. 1. To promote breadth, the generation prompt asks the model to span three coarse cognitive levels: perception (recognition or localization of a finding), characterization (description or grading of its imaging features), and diagnosis (integration of findings into a diagnostic conclusion). These levels serve only as prompt-level scaffolding and an initial coarse categorization; they are not used as prespecified strata for downstream analysis. Instead, downstream analyses use more granular clinical subcategory tags derived from question content and tailored to the relevant anatomy, imaging findings, and disease context. Each question undergoes structural validation and a report-grounded check confirming that the correct answer is explicitly supported by the source text; failures are discarded. The pipeline runs automatically with human review at only two checkpoints (schema confirmation and template approval). The report-grounded check removes factually unsupported answers but cannot fully eliminate residual language-model authoring bias, an inherent limitation of automated question generation.

### Models and inference

Five VLMs were selected to represent contemporary systems available through commercial APIs or open-weight releases, while spanning both general-purpose and medically adapted model classes, and were evaluated strictly zero-shot: Claude Opus 4.6 (released 5 February 2026), GPT-5.2 (released 11 December 2025), Qwen3-VL-30B, MedGemma1.0-27B, and MedGemma1.5-4B. Each model received the composite image or images, the question, and the answer options only, and never saw the radiology report. Closed-weights models were accessed through privacy-preserving institutional API endpoints and open-weights models were run on-premises, with no clinical text leaving the institution. Because closed-model serving stacks may evolve after release, these results should be interpreted as specific to the named model releases and evaluation period.

### Blind ablation and public/private comparison

To isolate visual from language-prior contributions, models were re-evaluated with the image replaced by a blank input, holding question text, options, and prompt fixed (Claude Opus 4.6, GPT-5.2, and MedGemma1.0-27B, reported in Table 2 and Table 3). For every (dataset, question type, model), the sighted and blind accuracies are computed on the intersection of questions that are valid and scored in both runs, so that Δ = sighted minus blind is a same-question contrast; this also removes any effect of runs that sampled different pool sizes. To test whether the brain visual signal reflects genuine reading or public-image familiarity, the identical pipeline was used to generate a fully private in-house brain cohort, compared directly with the public PDGM cohort (no subsampling; Table 3).

### Statistics and reproducibility

Accuracy is the fraction of valid model responses matching ground truth; invalid or unparsable responses are excluded. Interval estimates are our primary summaries; we additionally report multiplicity-controlled significance tests as a confirmatory robustness check (described below). Because answer pools mix 2- to 5-option items, per-cell chance varies; a random baseline is computed as the mean of 1/k across each pool’s choice-count distribution. Blind effects are reported as the point difference Δ, defined as sighted minus blind. All analyses are deterministic given the fixed seed. All uncertainty in this study is quantified by a single nonparametric, case-clustered bootstrap (B = 2,000 resamples; seed 42) that resamples cases, not questions, with replacement, so that intervals reflect generalization to new patients rather than to new questions from the same patients. The identical procedure is applied throughout Tables 1-3: to the marginal zero-shot accuracies (Table 1), and, for the sighted-versus-blind contrasts (Tables 2 and 3), to the paired per-question difference Δ = sighted - blind computed on the question set scored in both runs, with the same resampled case set applied to both arms so that the pairing is preserved. All reported intervals are 95% percentile intervals. In total we report 40 accuracy intervals (5 models × 4 cohorts × 2 question types; Table 1) and 36 paired-difference intervals (24 in Table 2; 12 in Table 3). These are pointwise, per-cell intervals and are not adjusted for multiplicity. They are therefore not simultaneous: the nominal 95% coverage applies to each interval individually and not to the family, and under repeated sampling roughly 5% of unadjusted intervals would be expected to exclude the true value by chance. The intervals are presented as descriptive measures of estimation precision, and no conclusion in this work is conditioned on any single interval attaining a significance threshold. Accordingly, we do not infer that two models differ because their marginal intervals fail to overlap, and we assess comparative patterns by their consistency across cohorts, question types, and models rather than by any single cell. As a confirmatory robustness check, and using the same case-clustered resampling at higher resolution (B = 10,000; seed 42), we additionally compute multiplicity-controlled tests, controlling the false discovery rate (FDR) within each of two families by the Benjamini–Hochberg procedure. Family 1 (40 tests) asks whether each Table 1 accuracy exceeds its per-question chance level (one-sided; statistic the mean of 1[correct] − 1/k over the model’s valid pool). Family 2 (30 unique image-effect tests, with the six public-brain cells shared by Tables 2 and 3 counted once) asks whether Δ differs from zero (two-sided percentile-inversion bootstrap p, consistent by construction with the reported 95% intervals); for these cells we also perform two one-sided equivalence tests (TOST) against the prespecified ± 0.05 margin, declaring equivalence when the 90% interval lies within ± 0.05. Per-cell p-values, Benjamini–Hochberg q-values and TOST outcomes for the image effect are given in Table 4; the accuracy-versus-chance family is reported in Supplementary Table 1. After FDR control, 37 of 40 accuracies exceed chance (the exceptions are Qwen3-VL-30B on liver MRI and MedGemma1.0-27B on brain RADS-style), and all 30 image-effect comparisons resolve: 17 show a genuine nonzero Δ and 16 are equivalent to zero within ± 0.05 (three are both), with the frontier models’ brain report-derived accuracy equivalent between sighted and blind (Claude Opus 4.6, GPT-5.2 and MedGemma1.0-27B all TOST q < 0.001).

### Reporting of AI and language-model use

Language models are used in this study in two roles: inside the benchmark pipeline to author report-derived questions (subject to the report-grounded validation above), and as the systems under evaluation. No language model is an author of this manuscript. Large language models were used to assist in editing the manuscript text; all content was reviewed, verified, and approved by the authors.

### Ethics

This study was approved by the Institutional Review Board of the University of California, San Francisco (UCSF IRB, Human Research Protection Program; protocols 22-37795 and 18-24775; https://irb.ucsf.edu), which granted a waiver of informed consent for retrospective research using electronic health record data. All methods were performed in accordance with the relevant guidelines and regulations, including the Declaration of Helsinki and the Health Insurance Portability and Accountability Act (HIPAA). All patient data remained within institutional secure infrastructure or approved HIPAA-compliant platforms. No clinical text was transmitted through public consumer-facing APIs.

## Supplementary Material

This is a list of supplementary files associated with this preprint. Click to download.

• SupplementarySR.docx

## Figures and Tables

**Figure 1:**
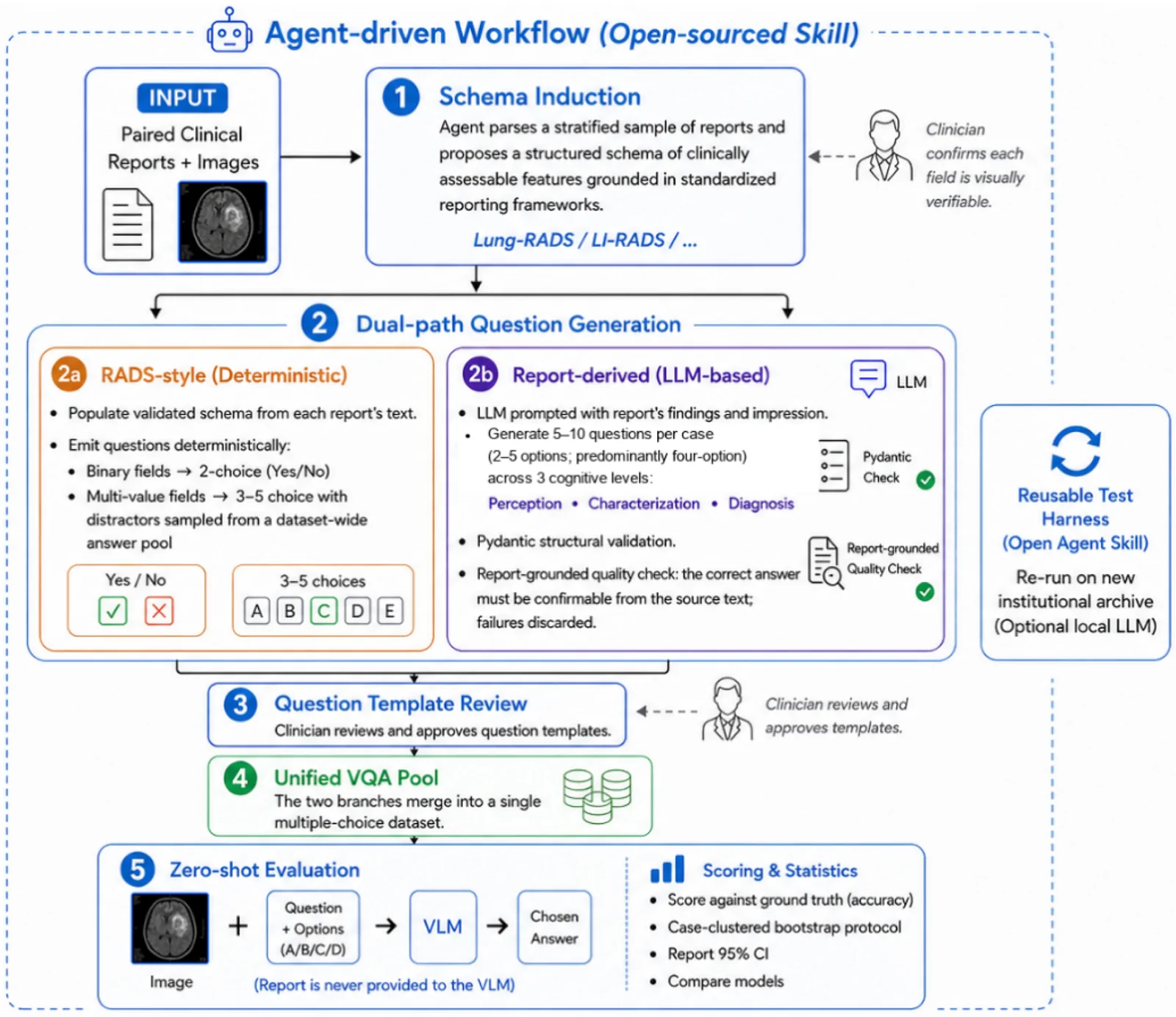
Automated, agent-driven pipeline for a contamination-controlled radiology VQA benchmark. A dual-path generation strategy turns paired private reports and 3D images into clinically grounded multiple-choice questions: schema-driven RADS-style questions populated deterministically from text, and report-derived questions written by a language model and verified against the source report, followed by clinician validation and zero-shot evaluation. Report-derived items contain two to five answer options, with four-option items predominating. The workflow is released as a reusable test harness for institutional re-deployment.

**Figure 2:**
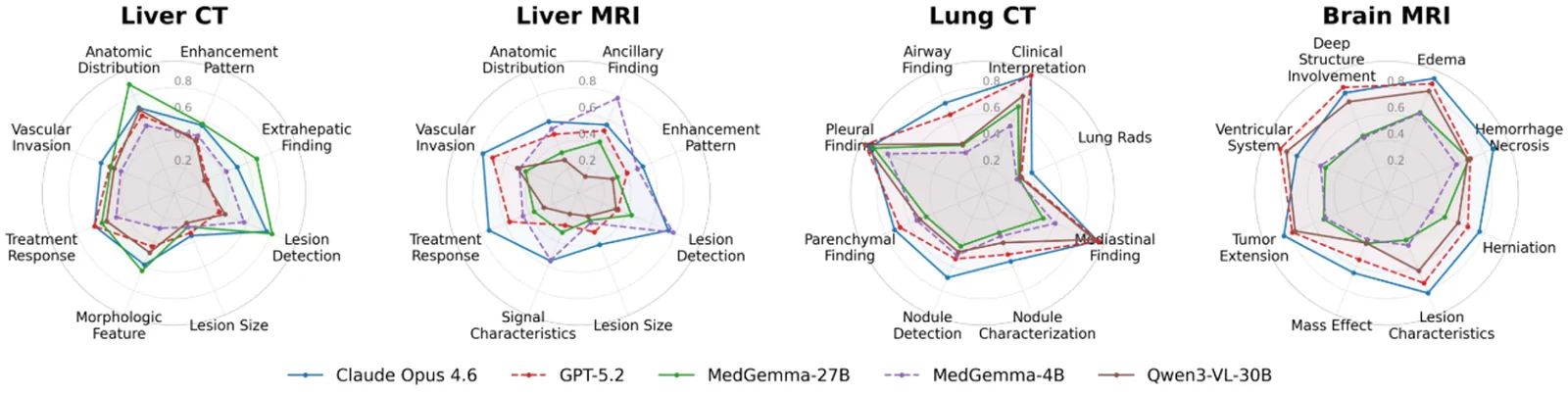
Per-subcategory accuracy on report-derived questions across the four datasets and five VLMs. Each panel is a polar plot whose axes are that dataset’s clinical subcategories; a longer radius is higher accuracy. Failures concentrate in quantitative and graded subcategories (for example lesion size, Lung-RADS category, and mass-effect grading), while qualitative recognition subcategories show comparatively higher accuracy.

**Table 1:** Zero-shot accuracy by dataset and question type. Values are mean ± half-width of the 95% case-clustered bootstrap confidence interval (B = 2000, seed 42). Highest point estimate per column within each block is in bold. *Brain MRI is the public PDGM cohort; a matched, fully private in-house brain cohort is compared in* Table 3.

Cases	Liver CT	Liver MRI	Lung CT	Brain MRI^a^
	998	531	500	480
Questions (RADS / Report-derived)	5978 / 8040	3569 / 5056	3085 / 3773	1817 / 2552
*(a) RADS-style questions*				
Random baseline	0.325	0.291	0.460	0.481
Claude Opus 4.6	0.453 ± 0.028	**0.571 ± 0.020**	0.563 ± 0.020	**0.666 ± 0.024**
GPT-5.2	0.406 ± 0.024	0.410 ± 0.020	0.516 ± 0.021	0.651 ± 0.022
MedGemma1.0-27B	**0.486 ± 0.010**	0.336 ± 0.016	0.525 ± 0.015	0.469 ± 0.023
MedGemma1.5-4B	0.425 ± 0.016	0.512 ± 0.018	0.512 ± 0.017	0.562 ± 0.023
Qwen3-VL-30B	0.481 ± 0.012	0.295 ± 0.016	**0.589 ± 0.016**	0.532 ± 0.025
*(b) Report-derived questions*				
Random baseline	0.316	0.319	0.312	0.297
Claude Opus 4.6	0.617 ± 0.020	**0.587 ± 0.019**	**0.698 ± 0.020**	**0.809 ± 0.016**
GPT-5.2	0.440 ± 0.019	0.386 ± 0.019	0.635 ± 0.022	0.752 ± 0.016
MedGemma1.0-27B	**0.645 ± 0.012**	0.348 ± 0.014	0.473 ± 0.015	0.466 ± 0.018
MedGemma1.5-4B	0.478 ± 0.015	0.549 ± 0.014	0.493 ± 0.015	0.474 ± 0.019
Qwen3-VL-30B	0.440 ± 0.011	0.278 ± 0.014	0.563 ± 0.014	0.673 ± 0.018

**Table 2:** Blind ablation. The three probed models receive questions and options with the image replaced by a blank input. Each model is scored on the question set shared by its sighted and blind runs, so Δ = sighted - blind is a same-question contrast. The prespecified ± 0.05 margin provides a descriptive reference for the magnitude of Δ; all CIs are pointwise and unadjusted for multiplicity. Δ is reported as the point estimate ± half-width of the 95% case-clustered paired bootstrap interval (B = 2000, seed 42), the same procedure as Table 1 applied to the paired per-question difference, with the same resampled case set used for the sighted and blind arms.

Dataset	Q. type	Claude Opus 4.6		GPT-5.2		MedGemma1.0-27B	
		Blind	Δ	Blind	Δ	Blind	Δ
Liver CT	RADS-style	0.524	−0.065 ± 0.046	0.364	+ 0.041 ± 0.027	0.384	+ 0.102 ± 0.014
Liver CT	Report-derived	0.609	+ 0.007 ± 0.025	0.392	+ 0.049 ± 0.020	0.355	+ 0.291 ± 0.015
Liver MRI	RADS-style	0.583	−0.021 ± 0.029	0.241	+ 0.157 ± 0.025	0.380	−0.044 ± 0.013
Liver MRI	Report-derived	0.597	−0.007 ± 0.019	0.263	+ 0.123 ± 0.021	0.347	+ 0.000 ± 0.007
Lung CT	RADS-style	0.726	−0.163 ± 0.032	0.531	−0.016 ± 0.018	0.452	+ 0.072 ± 0.013
Lung CT	Report-derived	0.680	+ 0.016 ± 0.022	0.666	−0.031 ± 0.016	0.432	+ 0.041 ± 0.011
Brain MRI	RADS-style	0.649	+ 0.017 ± 0.031	0.559	+ 0.092 ± 0.024	0.467	+ 0.002 ± 0.013
Brain MRI	Report-derived	0.811	−0.007 ± 0.021	0.743	+ 0.003 ± 0.020	0.474	−0.008 ± 0.013

**Table 3:** Public versus private brain MRI, matched generation. The same dual-question pipeline is applied to the public PDGM cohort and to a fully private in-house brain cohort; only the imaging source differs. Accuracies are same-question paired (sighted ∩ blind); Δ = sighted - blind. On report-derived questions the cohorts are indistinguishable; on RADS-style the frontier models score higher on public than private imaging, whereas MedGemma1.0-27B differs by < 0.01; the analysis does not establish the source of these cohort differences. Sighted and blind accuracies are paired on the shared valid-scored questions (sighted ∩ blind); because the blind report-derived runs were subsampled, this intersection is smaller than the full sighted pool used in Table 1, so the report-derived sighted values here differ from Table 1 by ≤ 0.007, whereas the RADS-style pools nearly coincide and match Table 1 to three decimals. Δ is reported as the point estimate ± half-width of the 95% case-clustered paired bootstrap interval (B = 2000, seed 42).

Cohort	Question type	Model	Sighted	Blind	Δ
PDGM (public)	RADS-style	Claude Opus 4.6	0.666	0.649	+ 0.017 ± 0.031
		MedGemma1.0-27B	0.469	0.467	+ 0.002 ± 0.013
		GPT-5.2	0.651	0.559	+ 0.092 ± 0.024
	Report-derived	Claude Opus 4.6	0.805	0.811	−0.007 ± 0.021
		MedGemma1.0-27B	0.467	0.474	−0.008 ± 0.013
		GPT-5.2	0.745	0.743	+ 0.003 ± 0.020
In-house (private)	RADS-style	Claude Opus 4.6	0.498	0.631	−0.133 ± 0.032
		MedGemma1.0-27B	0.478	0.455	+ 0.023 ± 0.016
		GPT-5.2	0.460	0.502	−0.042 ± 0.021
	Report-derived	Claude Opus 4.6	0.791	0.808	−0.017 ± 0.020
		MedGemma1.0-27B	0.437	0.461	−0.024 ± 0.014
		GPT-5.2	0.760	0.740	+ 0.020 ± 0.019

**Table 4:** Image effect under multiplicity control. Every unique (cohort, question type, model) blind comparison, with Δ = sighted - blind and its 95% case-clustered paired bootstrap interval; the two-sided bootstrap p that Δ ≠ 0 with its Benjamini-Hochberg q (Family 2, 30 tests); and two one-sided equivalence tests (TOST) against the prespecified ± 0.05 margin (B = 10,000; seed 42). Verdict: Effect = FDR-significant Δ ≠ 0; Equivalent = TOST-significant ∣Δ∣ < 0.05; Effect (small) = both, i.e. reliably nonzero but smaller than the margin. The six public-brain cells shared by Tables 2 and 3 are listed once. Shaded rows are the private in-house brain cohort.

Cohort	Q. type	Model	Δ (95% CI)	p / q (FDR)	TOST p / q	Verdict
Liver CT	RADS-style	Claude Opus 4.6	−0.065 [−0.114, −0.016]	0.010 / 0.018	0.730 / 0.996	Effect
Liver CT	RADS-style	GPT-5.2	+ 0.041 [+ 0.015, + 0.068]	0.003 / 0.006	0.265 / 0.397	Effect
Liver CT	RADS-style	MedGemma1.0-27B	+ 0.102 [+ 0.088, + 0.117]	< 0.001 / <0.001	1.000 / 1.000	Effect
Liver CT	Report-derived	Claude Opus 4.6	+ 0.007 [−0.018, + 0.032]	0.615 / 0.683	< 0.001 / 0.001	Equivalent
Liver CT	Report-derived	GPT-5.2	+ 0.049 [+ 0.028, + 0.069]	< 0.001 / <0.001	0.448 / 0.640	Effect
Liver CT	Report-derived	MedGemma1.0-27B	+ 0.291 [+ 0.275, + 0.305]	< 0.001 / <0.001	1.000 / 1.000	Effect
Liver MRI	RADS-style	Claude Opus 4.6	−0.021 [−0.050, + 0.007]	0.144 / 0.206	0.024 / 0.046	Equivalent
Liver MRI	RADS-style	GPT-5.2	+ 0.157 [+ 0.133, + 0.181]	< 0.001 / <0.001	1.000 / 1.000	Effect
Liver MRI	RADS-style	MedGemma1.0-27B	−0.044 [−0.058, −0.031]	< 0.001 / <0.001	0.212 / 0.353	Effect
Liver MRI	Report-derived	Claude Opus 4.6	−0.007 [−0.025, + 0.012]	0.497 / 0.596	< 0.001 / <0.001	Equivalent
Liver MRI	Report-derived	GPT-5.2	+ 0.123 [+ 0.103, + 0.143]	< 0.001 / <0.001	1.000 / 1.000	Effect
Liver MRI	Report-derived	MedGemma1.0-27B	+ 0.000 [−0.007, + 0.008]	0.939 / 0.939	< 0.001 / <0.001	Equivalent
Lung CT	RADS-style	Claude Opus 4.6	−0.163 [−0.195, −0.131]	< 0.001 / <0.001	1.000 / 1.000	Effect
Lung CT	RADS-style	GPT-5.2	−0.016 [−0.034, + 0.002]	0.095 / 0.143	< 0.001 / <0.001	Equivalent
Lung CT	RADS-style	MedGemma1.0-27B	+ 0.072 [+ 0.059, + 0.085]	< 0.001 / <0.001	0.999 / 1.000	Effect
Lung CT	Report-derived	Claude Opus 4.6	+ 0.016 [−0.006, + 0.037]	0.154 / 0.210	< 0.001 / 0.002	Equivalent
Lung CT	Report-derived	GPT-5.2	−0.031 [−0.046, −0.015]	< 0.001 / <0.001	0.008 / 0.017	Effect (small)
Lung CT	Report-derived	MedGemma1.0-27B	+ 0.041 [+ 0.031, + 0.052]	< 0.001 / <0.001	0.058 / 0.103	Effect
Brain MRI (public)	RADS-style	Claude Opus 4.6	+ 0.017 [−0.014, + 0.048]	0.283 / 0.355	0.018 / 0.036	Equivalent
Brain MRI (public)	RADS-style	GPT-5.2	+ 0.092 [+ 0.067, + 0.118]	< 0.001 / <0.001	0.999 / 1.000	Effect
Brain MRI (public)	RADS-style	MedGemma1.0-27B	+ 0.002 [−0.011, + 0.014]	0.822 / 0.850	< 0.001 / <0.001	Equivalent
Brain MRI (public)	Report-derived	Claude Opus 4.6	−0.007 [−0.028, + 0.015]	0.563 / 0.650	< 0.001 / <0.001	Equivalent
Brain MRI (public)	Report-derived	GPT-5.2	+ 0.003 [−0.017, + 0.022]	0.809 / 0.850	< 0.001 / <0.001	Equivalent
Brain MRI (public)	Report-derived	MedGemma1.0-27B	−0.008 [−0.021, + 0.006]	0.284 / 0.355	< 0.001 / <0.001	Equivalent
Brain MRI (in-house)	RADS-style	Claude Opus 4.6	−0.133 [−0.164, −0.102]	< 0.001 / <0.001	1.000 / 1.000	Effect
Brain MRI (in-house)	RADS-style	GPT-5.2	−0.042 [−0.064, −0.021]	< 0.001 / <0.001	0.233 / 0.368	Effect
Brain MRI (in-house)	RADS-style	MedGemma1.0-27B	+ 0.023 [+ 0.007, + 0.039]	0.005 / 0.009	< 0.001 / 0.001	Effect (small)
Brain MRI (in-house)	Report-derived	Claude Opus 4.6	−0.017 [−0.037, + 0.003]	0.096 / 0.143	< 0.001 / 0.001	Equivalent
Brain MRI (in-house)	Report-derived	GPT-5.2	+ 0.020 [+ 0.001, + 0.039]	0.046 / 0.076	0.002 / 0.004	Equivalent
Brain MRI (in-house)	Report-derived	MedGemma1.0-27B	−0.024 [−0.038, −0.010]	< 0.001 / 0.002	< 0.001 / <0.001	Effect (small)

## Data Availability

The in-house datasets facilitating the findings of this study are not openly accessible due to legitimate privacy and security concerns. The original EHR note data cannot be redistributed to researchers outside of those involved in the study, approved by the IRB at the principal institution. Access to anonymized data can be facilitated through a material transfer agreement (MTA) managed by the principal institution. Owing to privacy concerns, datasets generated and/or analyzed during this study are not publicly available but can be provided by the corresponding author upon a reasonable request. The public brain tumor MRI is available as the UCSF-PDGM dataset.

## Code availability

The benchmark-generation pipeline as an open agent skill with the analysis code that reproduces the reported statistics are available at https://github.com/Bol901/Med_VQA_Benchmark_Skill under Apache 2.0 license.
